# Determination of Insulator-to-Semiconductor Transition in Sol-Gel Oxide Semiconductors Using Derivative Spectroscopy

**DOI:** 10.3390/ma9010006

**Published:** 2015-12-23

**Authors:** Woobin Lee, Seungbeom Choi, Kyung Tae Kim, Jingu Kang, Sung Kyu Park, Yong-Hoon Kim

**Affiliations:** 1 SKKU Advanced Institute of Nanotechnology (SAINT), Sungkyunkwan University, Suwon 16419, Korea; 800mhz@naver.com (W.L.); oyenice@skku.edu (S.C.); 2 School of Electrical and Electronics Engineering, Chung-Ang University, Seoul 06974, Korea; 4444812@naver.com (K.T.K.); uangelion@gmail.com (J.K.); 3 School of Advanced Materials Science and Engineering, Sungkyunkwan University, Suwon 16419, Korea

**Keywords:** sol-gel oxide, derivative spectroscopy, insulator-to-semiconductor transition

## Abstract

We report a derivative spectroscopic method for determining insulator-to-semiconductor transition during sol-gel metal-oxide semiconductor formation. When an as-spun sol-gel precursor film is photochemically activated and changes to semiconducting state, the light absorption characteristics of the metal-oxide film is considerable changed particularly in the ultraviolet region. As a result, a peak is generated in the first-order derivatives of light absorption (*A′*) *vs.* wavelength (λ) plots, and by tracing the peak center shift and peak intensity, transition from insulating-to-semiconducting state of the film can be monitored. The peak generation and peak center shift are described based on photon-energy-dependent absorption coefficient of metal-oxide films. We discuss detailed analysis method for metal-oxide semiconductor films and its application in thin-film transistor fabrication. We believe this derivative spectroscopy based determination can be beneficial for a non-destructive and a rapid monitoring of the insulator-to-semiconductor transition in sol-gel oxide semiconductor formation.

## 1. Introduction

Recently, metal-oxide based electronics have gained a significant interest in flexible and transparent electronics owing to their outstanding electrical performance and high optical transparency [1,2,3]. Among several material candidates, amorphous indium-gallium-zinc oxide (IGZO) is regarded as one of the most promising material because of its excellent uniformity and relatively wide process window. Generally, the IGZO films are deposited by vacuum deposition using sputtering or atomic layer deposition, even at a low temperature [4,5]. In addition, solution-based deposition of IGZO films is also favorable for ambient deposition, low-temperature processing, and roll-to-roll fabrication of IGZO-based flexible electronics [6,7,8]. Moreover, according to the recent reports, the electrical performance of solution-processed oxide thin-film transistors (TFTs) can be enhanced comparable to those of sputter-deposited oxide TFTs, suggesting their potential application in high-performance electronics such as flat-panel TFT backplanes [9,10].

For the solution processing of IGZO thin films, a high-temperature thermal annealing above 300 °C [11] or photochemical activation [12] is necessary to convert the as-spun precursor film into a functional and dense semiconducting layer. During this process, a considerable amount of organic components are removed from the precursor film, as a result of hydrolysis and condensation reactions [13], and finally form a dense metal-oxygen-metal (M–O–M) network. Because of this sol-gel reaction, in which the film is converted from an insulator to a semiconductor, the optical property (light absorption) of the film is gradually changed and can be utilized to determine the degree of insulating-to-semiconducting state transition. In addition to conventional electrical characterization methods such as Hall effect measurement and device-level characterization, which are very effective in obtaining detailed electrical properties [14,15], the optical spectroscopy based monitoring can offer a rapid determination of insulating-to-semiconducting state transition in solution-processed metal-oxide semiconductors.

Here, we demonstrate a derivative spectroscopy based monitoring of insulating-to-semiconducting state transition in sol-gel-processed oxide semiconductors. By tracing the first-order derivatives of light absorbance (*A*) or transmittance (*T*) of annealing films, the transition from insulating-to-semiconducting states can be monitored from peak tracing. We discuss and explain the evolution of the optical properties of the annealing film and show its application in photochemically activated metal-oxide thin-film devices.

## 2. Results and Discussion

### 2.1. Insulator-to-Semiconductor Transition of Sol-Gel Oxide Semiconductors

**Figure 1:**
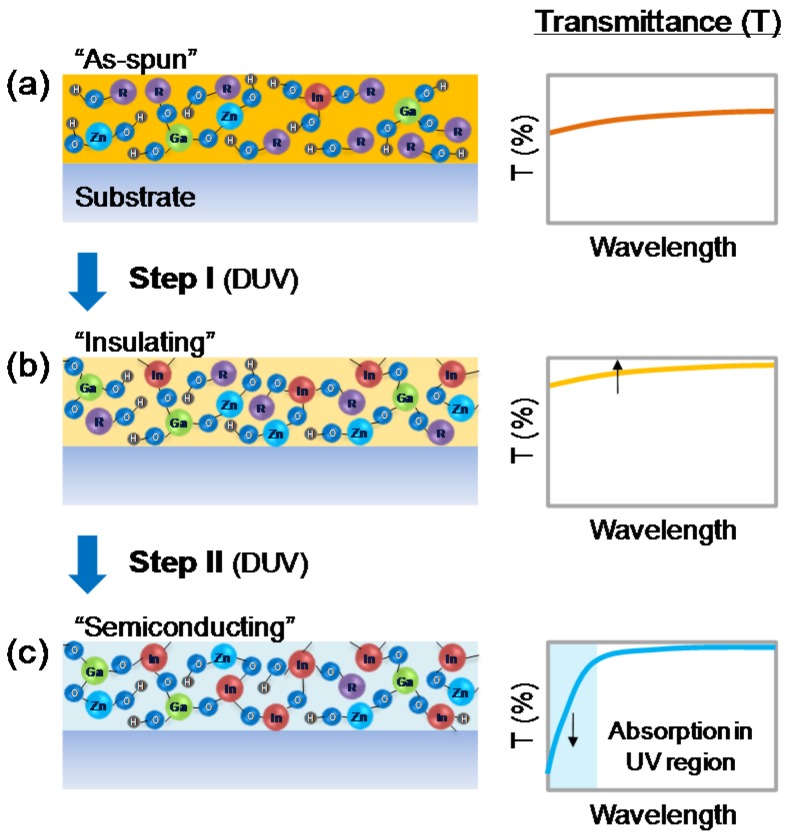
Schematics showing the evolution of a metal-oxide semiconductor film during the photochemical activation: (**a**) an as-spun film (insulating state); (**b**) after a short deep ultraviolet (DUV) irradiation (insulating state); and (**c**) after sufficient metal-oxygen-metal (M–O–M) network formation (semiconducting state).

The schematics shown in Figure 1 illustrate the evolution of a metal-oxide semiconductor film during photochemical activation process [12]. The as-spun film contains intermediate species from partial hydrolysis reaction and a significant amount of organic components including sol-gel reaction byproducts and residual solvent molecules. For this reason, the as-spun film shows non-negligible light absorption as shown in Figure 1a. Interestingly, after a short DUV irradiation for 5 min, the overall transmittance is increased which can be attributed to photochemical reaction partially removing organic components within the film (Figure 1b, Step I). Since the film is still in insulating state, a clear absorption edge is not observed in the measured wavelength range. Further irradiation of deep ultraviolet (DUV) (15–60 min), however, causes a prominent decrease of transmittance in the UV region and an absorption edge by forming a successful M–O–M network, as shown in Figure 1c (Step II). This particular change in the light absorption characteristics are expected in semiconductors having bandgaps near 3 eV (corresponding wavelength of ~413 nm), and thus can be utilized to trace the insulator-to-semiconductor transition during the film formation process. Also, it should be noted that if the as-spun film contains only a small amount of organic content inside, the transmittance change from the “as-spun film” state to “insulating film” state might be not significant. However, as will be shown later, even with a film having a thickness of a few tens of nanometers, the transmittance change is around 2%–5%, easily observable by UV/vis spectroscopy. Nonetheless, although the transmittance change from the “as-spun film” state to “insulating film” state is important, the transmittance change during the insulator-to-semiconductor transition is more important.

**Figure 2:**
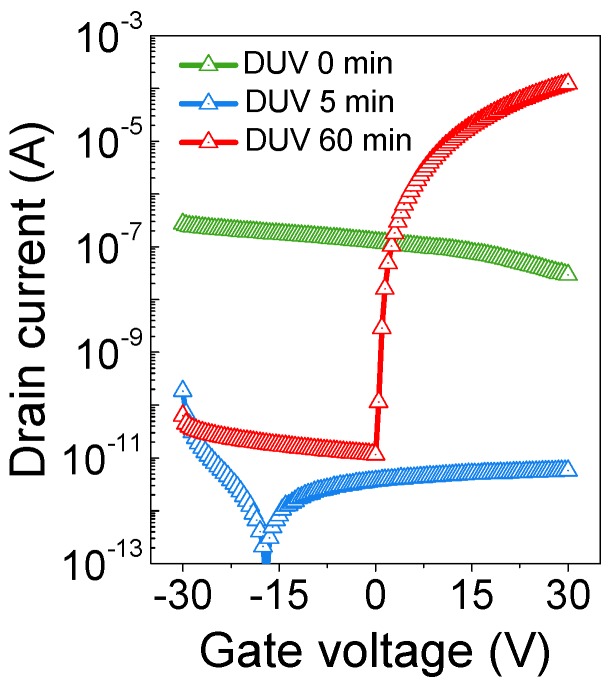
Variations in the transfer characteristics of indium-gallium-zinc oxide (IGZO) thin-film transistors (TFTs) with increasing DUV irradiation period. The TFTs were fabricated by using as-spun, 5-min DUV-annealed, and 60-min DUV-annealed IGZO channels (*V*_D_ = 30 V, *W*/*L* = 1000/70 µm).

In addition, the transition of the IGZO film from insulating to semiconducting states upon DUV irradiation can be verified by analyzing their electrical properties. To verify, IGZO TFTs with as-spun, 5-min DUV-annealed, and 60-min DUV-annealed IGZO channels were fabricated on Si/SiO_2_ substrates. Figure 2 shows corresponding transfer curves obtained from as-spun, 5-min DUV-annealed, and 60-min DUV-annealed IGZO TFTs. The as-spun film showed somewhat higher current level of *I*_DS_ compared to the 5-min DUV-annealed film, which can be attributed to possible ionic species present in the film. Then, with 5 min of DUV annealing, the current level decreases to 10^−11^–10^−12^ A, suggesting that the film is now in an electrically insulating state. Finally, with 60 min of DUV annealing, the film is in a semiconducting state, supporting the mechanism described in Figure 1. Also, it should be noted that the minimum occurred in the 5-min-annealed device is presumably due to the insulating property of the channel layer. Since the 5-min-annealed film is most likely an insulator, the source-to-drain current (*I*_DS_) level is very low, and therefore, the gate leakage current becomes noticeable in the transfer curve. Moreover, the gate electrode/gate insulator/channel (insulating)/source-drain electrode structure is similar to metal-insulator-metal (MIM) structure which exhibits MIM-like I–V characteristics as shown in Figure 2.

### 2.2. Determination of Insulator-to-Semiconductor Transition Using Derivative Spectroscopy

#### 2.2.1. Optical Characteristics of Sol-Gel Oxide Films

**Figure 3:**
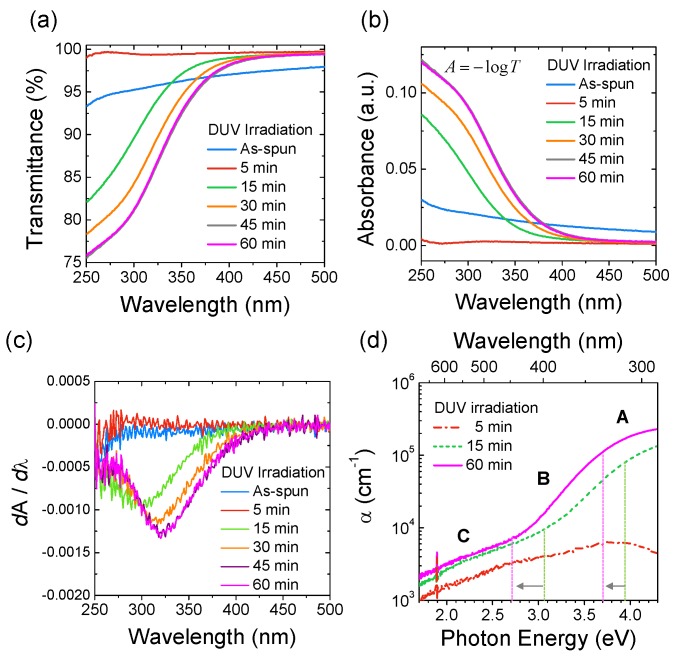
(**a**) Optical transmittance, and (**b**) absorbance data for as-spun and photo-activated IGZO films (DUV irradiation of 5–60 min). Here, the absorbance data were obtained from the transmittance data using an equation, *A* = –log*T*; (**c**) The first-order derivatives of absorbance (*A′*-λ); and (**d**) absorption coefficient (α) *vs.* photon energy (*E*) for photo-activated IGZO films.

Figure 3a,b shows the optical transmittance and absorbance data for as-spun and photo-activated IGZO films with different activation time, respectively. As shown here, the initial 5-min of DUV-irradiation increases the overall transmittance of the film from UV to visible light region. Then, significant light absorption occurs at λ < 400 nm after 15 min of DUV irradiation and with further irradiation, the light absorbance continuously increases and eventually saturates at around 45 min. After 45 min, the optical transmittance variation is negligible as shown by 45-min-annealed (grey color solid line) and 60-min-annealed IGZO films (pink-color solid line). Intuitively, the change in the optical transmittance or absorbance can be considered as parameters to determine the transition from insulating to semiconducting state. For instance, the transmittance value at wavelength of 300 nm changes from 95.2% (as-spun) to 99.4%, 84.3%, and 81.2% after 5 min, 30 min, and 60 min of irradiation, respectively. However, this approach is rather ambiguous since the transmittance data at specific wavelengths may overlap as shown in Figure 3a. A more reliable and feasible route to determine the transition from insulating to semiconducting state is to utilize the first-order derivatives of the light absorbance (*A′*). Previously the first-order derivatives of absorbance has been used in UV/vis spectroscopy to obtain the maximum absorbance wavelength at *A′* = 0, or to eliminate the effect of baseline shifts and baseline tilts which may occur during a spectroscopy observation [16]. However, it is found that the first-order derivatives of absorbance also can be used to determine the insulator-to-semiconductor transition. Figure 3c shows the first-order derivatives of absorbance (*A′*) *vs.* λ plots for as-spun and photo-activated IGZO films. The as-spun and 5-min-annealed films show nearly flat curves without any noticeable peaks, which are consistent with the *A*-λ plots. However, with a 15-min DUV irradiation, a peak appears in the *A′*-λ plot due to significant light absorption in the UV region. Also, upon further irradiation, a red-shift of the peak centers occurs in accordance with the shift observed in Figure 3a,b. Considering the amorphous nature of the IGZO films [12], the red-shift can be attributed to the change in the electronic structure of the IGZO film upon DUV irradiation.

Here, the significant light absorption in the UV region is attributed to the occurrence of semiconducting behavior in the IGZO film, having a bandgap around 3 eV. Also, the appearance of the peaks in the *A′*-λ plots, or the inflection points, can be explained based on the change in the photon-energy (*E*)-dependent absorption coefficient (α) for IGZO films. Figure 3d shows the α-*E* plots for 5, 15, and 60 min photo-activated IGZO films. In general, the α-*E* plot can be distinguished by three different regions of A, B and C, according to the α dependence on *E* [17]. Particularly, the three regions represent different optical transitions. In region A, the optical absorption follows Tauc relation, described by the equation, α*E* = *B(E* − *E_g_)*^2^, where *B* is material-dependent constant and *E_g_* the bandgap energy [17]. Rearranging this equation to α*/B* = *E* *+* *E_g_*^2^/*E* − 2*E_g_* shows the dependency of α on *E*. In contrast, in region B, the optical absorption follows Urbach rule, where α depends exponentially on the photon energy by α ~ *exp (E/E_u_)*, where *E_u_* is the Urbach energy [18]. Therefore, between the regions A and B, there is a clear change in the dependency of α on *E* which may lead to occurrence of an inflection point in the *A*-λ plot. In addition, as shown in Figure 3d, the absorption coefficient of the IGZO film increases with DUV irradiation time in the measured wavelength region. More importantly, the transition points, at which the α dependency on *E* changes, show a red-shift while increasing the DUV irradiation time. Along with the occurrence of an inflection point in the *A*-λ plot, this corresponds to the red-shift of the peak center observed in the *A′*-λ plots.

#### 2.2.2. Extraction of Peak Center (λ*_peak_*) and Peak Intensity (*I_peak_*)

**Figure 4:**
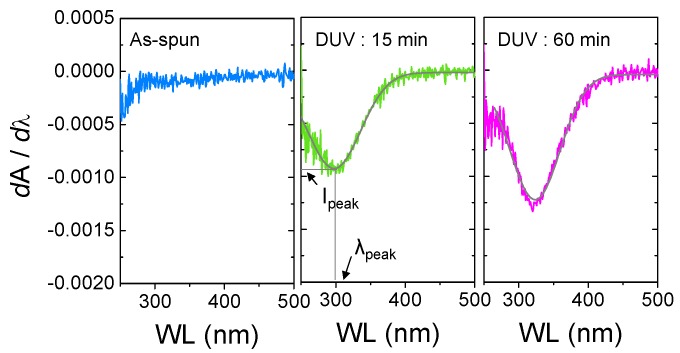
The first-order derivatives of absorbance *vs.* wavelength (*A′*-λ) and fitted curves of the photo-activated IGZO films, and acquisition of peak center (λ*_peak_*) and peak intensity (*I_peak_*).

**Figure 5:**
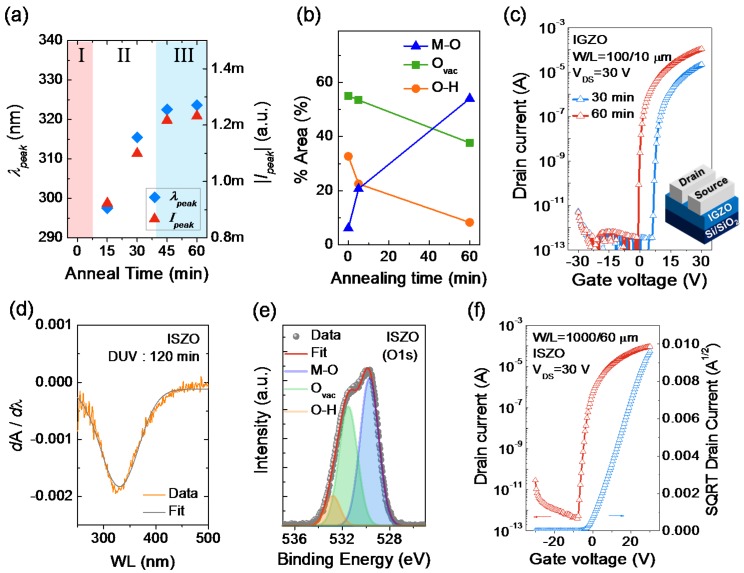
(**a**) The variation of peak center (λ*_peak_*) and peak intensity (*I_peak_*) as a function of photo-activation time: regime I—no peaks, regime II—increasing λ*_peak_* and *I_peak_*, and regime III—saturation of λ*_peak_* and *I_peak_*; (**b**) The changes in the binding states of oxygen atoms (represented as area %) in an IGZO film as a function of photo-activation time; (**c**) Transfer characteristics of IGZO TFTs with different photochemical activation time (30 min, 60 min); (**d**) *A′*-λ plot for the 120-min-annealed ISZO film; and (**e**) the O 1*s* peak from the XPS spectra of the ISZO film; (**f**) Transfer characteristics of photo-activated ISZO TFTs (120 min).

In the *A′*-λ plots, two important parameters can be extracted; the peak center (λ*_peak_*) and peak intensity (*I_peak_*) values. Particularly, the λ*_peak_* and *I_peak_* are obtained from the individual fitted curves as shown in Figure 4. Figure 5a summarizes the obtained λ*_peak_* and *I_peak_* values from the as-spun and photo-activated IGZO films. As displayed, three distinctive regimes are present: regime *I* with no observable peaks, regime II with increasing λ*_peak_* and *I_peak_*, and regime III with saturation of λ*_peak_* and *I_peak_*. Indeed, the regime I represents the insulating state of the IGZO film having a considerable amount of organic components and partial formation of M–O–M network. Correspondingly, the regimes II and III represent the progress and saturation of M–O–M network formation [12]. The progress of M–O–M network formation can be determined from the XPS analysis data as shown in Figure 5b, which represents the changes in the binding states of oxygen atoms in the IGZO films. Here, area % was obtained from the area ratios of the de-convoluted Gaussian peaks at binding energies of ~530, ~531, and ~532 eV, which represent oxygen atoms in oxide lattices without oxygen vacancies (M–O), in the vicinity of oxygen vacancies (M–O_vac_), and with hydroxide impurities (M–OH), respectively [19]. As shown in Figure 5b, the area % of M–O binding states is significantly increased with DUV annealing time. For the as-spun film, the area % of M–O binding states was only 6.1%, which implies that only a small portion of the film is constituted by the M–O–M network. After a short irradiation of DUV for 5 min, the area % of M-O binding states is increased to 20.7%; however, it was still comparably lower than those of M–O_vac_ (53.5%) and M–OH binding states (22.5%). Further increasing the annealing time to 60 min resulted in a significant increase in the area % of M-O binding states up to 54.0%.

#### 2.2.3. Correlation with TFT Performance

To find a correlation between the λ*_peak_* and *I_peak_* values and the actual electrical performance of the device, IGZO TFTs with 30-min and 60-min DUV-activated IGZO channels were fabricated. Figure 5c shows the transfer characteristics of IGZO TFTs with increasing photo-activation time. The 30-min photo-activated IGZO TFT, which has unsaturated λ*_peak_* and *I_peak_* values, exhibited a relatively low field-effect mobility of 0.77 cm^2^/Vs, positive turn-on voltage of ~6 V, and threshold voltage (*V*_TH_) of 11.2 V. In contrast, the 60-min photo-activated TFT, which has saturated λ*_peak_* and *I_peak_* values, showed an improved field-effect mobility of 2.08 cm^2^/Vs, near-zero turn-on voltage, and *V*_TH_ of 4.1 V. The enhanced mobility as well as the negative *V*_TH_ shift towards zero-volt can be attributed to denser and closely packed IGZO film from prolonged photo-activation, which provides sufficient overlap of the s-orbitals of metal ions and enhanced electron conduction, as observed in post-annealed zinc-tin-oxide TFTs under a wet-air environment [20]. In addition, the chemical bonding states within the film and the degree of M–O–M network formation can also affect their electrical properties [21]. Nonetheless, the λ*_peak_* and *I_peak_* values obtained from the *A′*-λ plots can be correlated with the actual performance of the device along with for the determination of the semiconductor formation.

To confirm the validity of the spectral determination method in other oxide materials system, ISZO thin films and TFTs were fabricated and their optical, chemical, and electrical properties were characterized. Figure 5d shows the *A′*-λ plot for a 120-min-annealed indium-gallium-zinc oxide (ISZO) film. Likewise, a clear peak is observed in the *A′*-λ plot having λ*_peak_* and |*I_peak_*| values of 330.1 nm and 0.0018, respectively. Also, Figure 5e shows the O 1*s* peak from the XPS spectra of the photo-activated ISZO film. A successful M–O–M network formation is confirmed having area % of 50.6%, 41.4% and 8.3% for M–O, M–O_vac_ and M–OH binding states, respectively. The transfer characteristics of ISZO TFTs further shows that the photo-activated ISZO films exhibit semiconducting behavior having a field-effect mobility of 1.64 cm^2^/Vs (Figure 5f). From these results, it is understood that there is a correlation between the λ*_peak_* and *I_peak_* values and the actual electrical performance of the transistors. Therefore, we believe that the derivative spectroscopy based determination of the sol-gel oxide semiconductors can be an efficient and rapid way to monitor the semiconductor formation.

## 3. Materials and Methods

As a material platform, a well-known material system, IGZO was selected. Initially, an IGZO precursor solution was prepared by dissolving 0.085 M of indium nitrate hydrate, 0.0125 M of gallium nitrate hydrate, and 0.0275 M of zinc acetate dehydrate (In:Ga:Zn = 6.8:1:2.2) in 2-methoxyethanol (all purchased from Sigma-Aldrich (St. Louis, MO, USA)). The total concentration of the precursor solution was 0.125 M. After a thorough stirring for up to 12 h, the IGZO precursor solution was spin-coated on a glass substrate or on a heavily doped silicon wafer having a 200-nm-thick thermally grown SiO_2_ film. Afterwards, the spun IGZO film was photochemically activated for 5–60 min in N_2_-rich atmosphere using a UV/ozone treatment system (UV253H, Filgen, Nagoya, Japan, intensity of ~28 mW/cm^2^). The thickness of the IGZO film after 60 min of photochemical activation was around 10 nm which was measured by using an alpha-step surface profiler (P-10, KLA Tencor, Milpitas, CA, USA). After patterning the IGZO channel by photolithography and wet etching, indium-zinc oxide or aluminum source/drain electrodes were deposited by sputtering or thermal evaporation, and the channel width and length of the thin-film transistors (TFTs) were 100–1000 µm and 10–60 µm, respectively. In addition, another oxide-based material system, such as ISZO films were also prepared, using strontium nitrate as a precursor, to evaluate the derivative spectroscopy in other material systems. The total concentration of the ISZO precursor solution was 0.119 M and the corresponding In:Sr:Zn ratio was 6.8:0.5:2.2.

The optical transmittances of as-spun and photochemically activated oxide films were analyzed by UV/vis spectrophotometer (Agilent 8453, Agilent Technologies, Santa Clara, CA, USA) in a wavelength range of 250–500 nm. The absorbance was calculated using the transmittance data with an equation, *A* = −log*T*. The obtained absorbance data was then differentiated with respect to wavelength (*A′*). From the *A′*-λ plots, peak intensity (*I_peak_*) and peak center (λ*_peak_*) were identified from fitted curves. Also, the absorption coefficient (α) of IGZO films were obtained using the equation, α = −(1/*d*)∙ln(*I*/*I*_0_), where *d* is the thickness of film, *I* transmitted light intensity and *I*_0_ incident light intensity. In addition, to determine the atomic bonding states of metal-oxide films, we carried out X-ray photoelectron spectroscopy (XPS, ESCA 2000, VG Microtech, East Grinstead, UK) analysis for IGZO and ISZO films. The electrical characteristics of the TFTs were measured by using a semiconductor parameter analyzer (Agilent 4155C, Agilent Technologies, Santa Clara, CA, USA) in dark ambient condition.

## 4. Conclusions

In this paper, we demonstrate a derivative spectroscopy to determine insulator-to-semiconductor transition in sol-gel metal-oxide semiconductors. It is found that the first-order derivatives of optical absorbance can be utilized to determine the transition by tracing a peak evolution in the *A′*-λ plots. We believe that this spectral determination method can be beneficial as a non-destructive and a rapid way to monitor the insulator-to-semiconductor transition during the fabrication of oxide semiconductors.
